# Burden of Cancers Attributable to Infectious Agents in Nigeria: 2012–2014

**DOI:** 10.3389/fonc.2016.00216

**Published:** 2016-10-24

**Authors:** Michael Odutola, Elima E. Jedy-Agba, Eileen O. Dareng, Emmanuel Aja Oga, Festus Igbinoba, Theresa Otu, Emmanuel Ezeome, Ramatu Hassan, Clement A. Adebamowo

**Affiliations:** ^1^Institute of Human Virology, Abuja, Nigeria; ^2^Department of Non-communicable Disease Epidemiology, London School of Hygiene and Tropical Medicine, London, UK; ^3^Department of Public Health and Primary Care, Center for Cancer Genetic Epidemiology, University of Cambridge, Cambridge, UK; ^4^Battelle Memorial Institute, Baltimore, MD, USA; ^5^National Hospital Abuja, Abuja, Nigeria; ^6^University of Abuja Teaching Hospital Gwagwalada, Gwagwalada, Nigeria; ^7^University of Nigeria Teaching Hospital Enugu, Enugu, Nigeria; ^8^Federal Ministry of Health, Abuja, Nigeria; ^9^Department of Epidemiology and Public Health, Marlene and Stewart Greenebaum Comprehensive Cancer Center, Institute of Human Virology, University of Maryland School of Medicine, Baltimore, MD, USA

**Keywords:** cancers, infectious agents, population-attributable fraction, cancer registry, Nigeria

## Abstract

**Introduction:**

Infections by certain viruses, bacteria, and parasites have been identified as risk factors for some cancers. In Nigeria, like many other developing countries, infections remain a leading cause of morbidity and mortality. While there are data on the incidence of different cancers in Nigeria, there has been no study of cancers attributable to infections. This study was carried out to determine the burden of cancers attributable to infections using data from two population-based cancer registries (PBCRs) in Nigeria.

**Methods:**

We obtained data on cancers associated with EBV, human papillomavirus (HPV), hepatitis B and C, HIV, HHV8, *Helicobacter pylori*, and *Schistosoma* spp. from the databases of Abuja and Enugu cancer registries in Nigeria. We used population-attributable fraction for infections-associated cancers in developing countries that are based on prevalence data and relative risk estimates from previous studies.

**Results:**

The PBCRs reported 4,336 incident cancer cases [age standardized incidence rate (ASR) 113.9 per 100,000] from 2012 to 2014, of which 1,627 (37.5%) were in males and 2,709 (62.5%) were in females. Some 1,030 (23.8%) of these cancers were associated with infections (ASR 44.4 per 100,000), while 951 (22.0%) were attributable to infections (ASR 41.6 per 100,000). Cancers of the cervix (*n* = 392, ASR 28.3 per 100,000) and liver (*n* = 145, ASR 3.4 per 100,000); and non-Hodgkin’s lymphoma (*n* = 110, ASR 2.5 per 100,000) were the commonest infections-associated cancers overall. The commonest infectious agents associated with cancers in this population were HPV, EBV, hepatitis B and C, HIV, and HHV8.

**Conclusion:**

Our results suggest that 23.8% of incident cancer cases in this population were associated with infections, while 22.0% were attributable to infections. The infections attributable cancers are potentially preventable with strategies, such as vaccination, risk factor modification, or anti-infective treatment.

## Introduction

Infectious agents are important contributors to the global cancer burden particularly in Africa. Of the 12.7 million new cancer cases that were diagnosed worldwide in 2008, about two million (16.1%) were attributable to infectious agents, of which 1.6 million occurred in less developed regions of the world (1). In its 2009 review of the carcinogenicity of infectious agents, the International Agency for Research on Cancer (IARC) found sufficient evidence to conclude that seven viruses: human papillomavirus (HPV), Epstein–Barr virus, hepatitis B virus (HBV), hepatitis C virus (HCV), human immunodeficiency virus type-1 (HIV-1), Kaposi’s sarcoma herpes virus (KSHV), and human T-cell leukemia virus type-1 (HTLV-1), the bacterium *Helicobacter pylori*, and the parasites *Clonorchis sinensis (C. sinensis), Opisthorchis viverrini (C. sinensis)*, and *Schistosoma haematobium* (*S. haematobium)* were carcinogenic to humans (2).

The proportion of cancers attributable to infections in developing countries has been estimated to vary between 20 and 30% which contrasts with 3–10% in developed countries (1, 3–5). Although there are estimates of the burden of infection-associated cancers based on cancer registry data for countries, such as Australia (3), United Kingdom (4), Netherlands (5), United States (6), South Korea (7), France (8), and China (9), there are no country-specific estimates for countries in Africa, including Nigeria. It is important to quantify the proportion of the cancer burden that could be prevented by the implementation of effective interventions that limit exposure to the infections-associated with cancers, particularly in Africa.

Since the resuscitation and strengthening of cancer registration in Nigeria starting 2009, more population-based cancer registries (PBCRs) have become operational (10–12). The aim of this study is to estimate the burden of cancers attributable to infections in Nigeria between 2012 and 2014 from these PBCRs.

## Materials and Methods

### Data Sources

We retrieved cancer incidence information from two PBCRs in Nigeria, the Abuja and Enugu cancer registries, for the period 2012–2014. The Abuja Cancer Registry (ABCR), established in 2009, has a catchment area that covers the entire federal capital territory and a population of 1,406,239 people (10). The Enugu Cancer Registry (ECR) was established in 2012 and covers an area around the greater Enugu city metropolis with a population of 1,103,153 people (12). The registries utilize the International Classification of Disease for Oncology, 3rd Edition (ICD-O3) for coding and classification of cancers. The ABCR uses CanReg4 software for storing and processing data, while the ECR uses CanReg5. Approval for cancer registration activities and research was obtained from the National Health and Research Ethics Committee of Nigeria (NHREC). Anonymity and confidentiality were maintained in all analyses and publications derived from the cancer registry data.

### Data Handling and Statistical Analysis

Data checks were done by Michael Kolawole Odutola and Elima E. Jedy-Agba using the IARC CanReg5 software. We performed quality control checks to remove duplicates, and to ensure logical correctness and overall accuracy of the data. Analysis of incident cancer cases and age standardized incidence rate (ASR) calculation was generated by the CanReg5 software.

For this analysis, we retrieved age and sex-specific number of incident cancers reported by the cancer registries during the period under study. Using the classification of carcinogens by IARC in monograph 100b, we identified the following cancers considered to be associated with infectious agents in humans: nasopharygeal (C11), oropharyngeal (C01, C09, C10), stomach (C16), liver (C22), bladder (C67), Hodgkin lymphoma (HL; C81), non-Hodgkin lymphoma (NHL; C82–C85; C96), Kaposi Sarcoma (KS) (C46), and anal cancers (C21) in both sexes; cervical (C53), vulvar (C51), and vaginal cancers (C52) in females; and penile cancer (C60) in males (Table 1). We considered all the infectious agents identified by IARC as Group 1 carcinogens, except Human T-cell leukemia virus type-1 (HTLV-1); *C. sinensis* and *O. viverrini* associated with acute T-cell leukemia/lymphoma and cancer of the bile duct, respectively, because of the rarity of these cancers in African populations (13).

**Table 1:** **List of group 1 carcinogenic biological agents and related cancers**.

Infections-associated cancers	Group 1 agents
Cervix	Human papillomavirus
Vulva	Human papillomavirus
Vagina	Human papillomavirus
Anus	Human papillomavirus
Oropharynx	Human papillomavirus
Bladder	*Schistosoma haematobium*
Liver	Hepatitis B virus/hepatitis C virus
Nasopharynx	Epstein–Barr virus
Stomach	*Helicobacter pylori*
Non-Hodgkin lymphoma	Epstein–Barr virus/human immunodeficiency virus type-1
Hodgkin lymphoma	Epstein–Barr virus
Kaposi sarcoma	Human herpesvirus 8/human immunodeficiency virus type-1
Penis	Human papillomavirus

We used the standard formula for population-attributable fraction (PAF) (4):

PAF=p(r−1)/[p(r−1)+1]

where *r* represents the relative risk of exposure and *p* its prevalence in the population. The application of this formula requires information about the prevalence of the exposure to the infectious agents in the population, as well as the corresponding relative risks. In previous studies, this method was used to estimate the number of cancers attributable to HBV, HCV, *H. pylori*, and HIV. For HPV and EBV, the PAF was assumed to be equivalent to the prevalence of viral DNA in the tumor cells.

In this study, we used the PAF for infectious agents associated with cancers that are derived from prevalence and risk estimates from developing countries (Table 2). We did not have PAF from Nigeria because of the absence of rigorous and robust measures of relative risk for the different infectious agents in this population. Additionally, data on prevalence of exposure to most of the infectious agents is scanty. The few prevalence studies from Nigeria had relatively small sample sizes (less than 50) and were not population based. For each infectious agent, we estimated the number of new cancer cases attributable to that infection by multiplying the overall numbers of cancer cases reported per cancer site with the PAF estimates extracted from meta-analyses.

**Table 2:** **Previous prevalence studies on infectious agents and cancer**.

Infectious agent	Cancer site	ICD-O code	Prevalence of infectious agents in cancers (SSA population) (%)	Studies in African populations	PAF estimates used in this study %
HPV	Cervix	C53	83–98	Nigeria, South-Africa (14), Ghana (15)	100 (16)
	Vulva	C51	100	Botswana (17)	40 (18)
	Vagina	C52	–	None	40–70 (13, 18)
	Anus	C21	88	Nigeria, Mali, Senegal (19)	88–90 (13, 19)
	Oropharynx	C01–C02; C09–C10	19	Ghana (20)	12–46 (13, 21)
	Penis	C60	68	Kenya (22)	40–48 (13, 23)
Shistosoma	Bladder	C67	–	None	56 (13)
HBV/HCV	Liver	C22	74–77	Nigeria (24), Gambia (25)	92 (13)
*H. pylori*	Stomach	C16	56–86	Nigeria (26), Uganda (27)	74 (13)
EBV	Nasopharynx	C11	–	None	100 (13, 28)
	HL	C81	55	Nigeria (29)	80 (13)
EBV/HIV	NHL	C82–C85; C96	80–95	Nigeria (29), Uganda (30)	100 (13)
HIV/HHV8	KS	C46	83	Nigeria, Benin, Côte d’Ivoire, Togo (31)	100 (32, 33)

In determining PAF estimates for cervical cancer, we identified other studies from Africa that reported the prevalence of HPV in cervical tumor cells to be between 83 and 98% (14, 15). However, we used a PAF of 100% because it is established that HPV is a necessary cause of cervical cancer (16, 34). For vulvar cancer, we found a Botswanan study with a sample size of 35 which reported an HPV prevalence of 100% (17). However, we used a PAF estimate of 40% obtained from meta-analyses for developing countries, because of the small sample size used in that study (13). We did not find any African regional study on the prevalence of HPV in vaginal cancer, hence, we used a PAF estimate of 40–70% from meta-analyses of studies from developing countries (13, 18). Because we did not find any individual study on HPV prevalence in anal cancer from Africa, we used a PAF estimate of 88–90% from meta-analyses that included cases from developing countries, including Nigeria, Mali, and Senegal (13, 19). A study from Ghana showed HPV prevalence of 16% in etiology of oropharyngeal cancer (OPC) (20), while a study evaluating HPV prevalence in oropharyngeal, and other head and neck cancers in Nigeria had suboptimal sample size and poor tissue sample quality (35); hence, we used PAF estimate of 12–46% from meta-analyses of studies from developing countries (13, 21). The prevalence of HPV in 22 penile cancer cases from Kenya was found to be 68.2% (22). As a result of the small sample size, we used a worldwide estimated PAF of 40–48% from meta-analyses (13, 23).

We used a PAF estimate of 56% for bladder cancer associated with *S. haematobium* from a meta-analysis for West-African countries (13). Although, we identified two studies from Nigeria and Gambia that reported the prevalence of HBV and HCV in liver cancer to range from 74 to 77% (24, 25), we used a PAF estimate of 92% from a meta-analysis of studies from developing countries (13). Two studies from Nigeria and Uganda evaluated the prevalence of *H. pylori* in stomach cancer to be 72–87% (26, 27); however, we used an estimated PAF of 74% from a meta-analysis of studies from developing countries (13). We did not find any studies of the prevalence of EBV in nasopharyngeal cancer in SSA, we used a PAF estimate of 100% from a meta-analysis of studies from developing countries (13, 28). For NHL in HIV infections, although we identified two studies from Nigeria and Uganda with prevalence ranging from 80 to 95% (29, 30), we used an estimated PAF of 100% based on meta-analysis of studies from developing countries because of the limited sample size of 44 used in the Nigerian study (13). The prevalence of EBV infection in Hodgkin’s Lymphoma from a Nigerian study was 55% (29) but the small sample size (11) was small so we used estimated PAF of 80% derived from a meta-analysis of studies from developing countries (13). HHV8 is recognized as a necessary cause of KS in HIV infections; therefore, we used PAF of 100% for KS (31–33).

We carried out sensitivity analyses for cancers attributable to infections, using GLOBOCAN 2012 database for Nigeria. GLOBOCAN 2012 database contains incidence estimates for only 7 (cancer of the liver, cervix, nasopharynx, bladder; HL, NHL; and KS) out of the 12 infections-associated cancers that we evaluated in our analyses.

## Results

### Overview

Over a 3-year period from 2012 to 2014, the Abuja and Enugu PBCRs reported 4,336 cancer cases [1,627 (37.5%) in males, 2,709 (62.5%) in females]. In total, 1,030 (23.8%) cancer cases were associated with infections (Table 3). The ASR of infections-associated cancers was 44.4 per 100,000. Approximately 90% (328 of 365) and 94% (623 of 665) of these infections-associated cancers were attributable to infections in males and females, respectively (Table 4). Of all the infections-associated cancers reported, 22.0% (951 of 4,336) were attributable to infections. The ASR for infections-attributable cancers was 41.6 per 100,000. Cancers attributable to infections constituted 20.2% (328 of 1,627) of cases in males and 23.0% (623 of 2,709) in females (Table 4).

**Table 3:** **Total number of cancer cases associated with infections in Nigeria from 2012 to 2014**.

Sex	Cancer site	ICD-O code	No. of cancer cases	% of total cancer	ASR
Female	Cervix	C53	392	30.0	28.3
	Vulva	C51	25	1.8	1.3
	Vagina	C52	8	0.6	0.4
	Anus	C21	17	1.3	0.7
	Oropharynx	C01–C02; C09–C10	8	0.6	0.5
	Bladder	C67	14	1.1	1.1
	Liver	C22	52	3.9	2.7
	Nasopharynx	C11	34	2.5	1.5
	Stomach	C16	23	1.7	1.4
	NHL	C82–C85; C96	49	3.7	2.3
	HL	C81	17	1.2	0.5
	KS	C46	26	2.0	1.1
Male	Penis	C60	2	0.2	0.1
	Anus	C21	21	2.7	1.4
	Oropharynx	C01–C02; C09–C10	15	1.9	0.8
	Bladder	C67	30	3.8	1.4
	Liver	C22	93	11.9	4.0
	Nasopharynx	C11	47	6.1	1.7
	Stomach	C16	33	4.4	1.7
	NHL	C82–C85; C96	61	7.6	2.7
	HL	C81	28	8.7	1.0
	KS	C46	35	4.6	1.3
Total			1,030	23.8	44.4

**Table 4:** **Population-attributable fraction (PAF) and estimated numbers of cancers attributable to infectious agents in Nigeria from 2012 to 2014**.

Infectious agent	Cancer site	ICD-O3 code	No. of cancer cases	ASR	PAF %	Cancer cases attributable to infections	ASR
**Female**							
HPV	Cervix	C53	392	28.3	100	392	28.3
	Vulva	C51	25	1.3	40	10	0.5
	Vagina	C52	8	0.4	40–70	3–6	0.2–0.3
	Anus	C21	17	0.7	88–90	15	0.6
	Oropharynx	C01–C02; C09–C10	8	0.5	12–46	1–4	0–0.3
HBV/HCV	Liver	C22	52	2.7	92	48	2.5
EBV	Nasopharynx	C11	34	1.5	100	34	1.5
	HL	C81	17	0.5	80	14	0.4
EBV/HIV	NHL	C82–C85; C96	49	2.3	100	49	2.3
HIV/HHV8	KS	C46	26	1.1	100	26	1.1
*H. pylori*	Stomach	C16	23	1.4	74	17	1.0
Schistosoma	Bladder	C67	14	1.1	57	8	0.6
Total (% of female cancers)			665 (25.0%)	41.8		623 (23.0%)	39.0–39.4
**Male**							
HPV	Penis	C60	2	0.1	40–48	1	0.1
	Anus	C21	21	1.4	88	18	1.2
	Oropharynx	C01–C02; C09–C10	15	0.8	12	2–7	0.1–0.4
HBV/HCV	Liver	C22	93	4.0	92	86	3.7
EBV	Nasopharynx	C11	47	1.7	100	47	1.7
	HL	C81	28	1.8	80	22	1.4
EBV/HIV	NHL	C82–C85; C96	61	2.7	100	61	2.7
HIV/HHV8	KS	C46	35	1.3	100	35	1.3
*H. pylori*	Stomach	C16	33	1.7	74	24	1.2
Schistosoma	Bladder	C67	30	1.4	57	17	0.8
Total (% of male cancers)			365 (22.4%)	16.9		328 (20.2%)	14.2–14.5
**Total (% of cancers in both sexes)**			1,030 (23.8%)	44.4		951 (22.0%)	41.2–41.6

In 2012, GLOBOCAN estimated that 102,079 [64,709 (63.3%) in women, 37,370 (36.6%) in men] cancer cases occurred in Nigeria. In total, 35.9% were associated with infections. The ASR for infections-associated cancers in Nigeria from the GLOBOCAN 2012 database was 50.0 per 100,000. Of all the infections-associated cancers reported, 33.8% were attributable to infections. The ASR for infections-attributable cancers in GLOBOCAN 2012 was 47.4 per 100,000 (Table 5).

**Table 5:** **Sensitivity analyses of cancers attributable to infectious agents in Nigeria from GLOBOCAN 2012 database**.

Infectious agent	Cancer site	ICD-O3 code	No. of cancer cases	ASR	PAF %	Cancer cases attributable to infections	ASR
**Female**							
HPV	Cervix	C53	14,089	29.0	100	14,089	29.0
	Vulva	C51	NA	NA	NA	NA	NA
	Vagina	C52	NA	NA	NA	NA	NA
	Anus	C21	NA	NA	NA	NA	NA
	Oropharynx	C01–C02; C09–C10	NA	NA	NA	NA	NA
HBV/HCV	Liver	C22	4,172	8.1	92	3,838	7.5
EBV	Nasopharynx	C11	406	0.6	100	406	0.6
	HL	C81	345	0.5	80	276	0.4
EBV/HIV	NHL	C82–C85; C96	1,778	2.8	100	1,778	2.8
HIV/HHV8	KS	C46	635	0.8	100	635	0.8
*H. pylori*	Stomach	C16	1,014	2.0	74	750	1.5
Schistosoma	Bladder	C67	523	0.7	57	298	0.4
Total (% of female cancers)			22,962 (35.5%)	44.5		22,084 (34.1%)	43.0
**Male**							
HPV	Penis	C60	NA	NA	NA	NA	NA
	Anus	C21	NA	NA	NA	NA	NA
	Oropharynx	C01–C02; C09–C10	NA	NA	NA	NA	NA
HBV/HCV	Liver	C22	7,875	15.0	92	7,245	13.8
EBV	Nasopharynx	C11	656	1.1	100	656	1.1
	HL	C81	559	0.8	80	447	0.6
EBV/HIV	NHL	C82–C85; C96	2,328	3.7	100	2,328	3.7
HIV/HHV8	KS	C46	812	1.3	100	812	1.3
*H. pylori*	Stomach	C16	875	2.0	74	648	1.5
Schistosoma	Bladder	C67	574	1.3	57	327	0.7
Total (% of male cancers)			13,679 (36.6%)	25.2		12,463 (33.4%)	22.7
**Total (% of cancers in both sexes)**			36,641 (35.9%)	50.0		34,547 (33.8%)	47.4

*NA means data not available*.

### Cervical Cancer and HPV

Cervical cancer was the commonest infections-associated cancer reported by ABCR and ECR within the study period. A total of 392 cases were reported, representing 30% of all cancers in females (Table 3). The ASR for cervical cancer was 28.3 per 100,000 (Table 3). The highest numbers of cervical cancer cases were seen in the 45 to 54 years age groups (110 cases), and ages 65 years and above (98 cases). Using a PAF of 100% for cervical cancer cases, we conclude that all 392 cervical cancer cases reported within the study period were attributable to HPV infection, with attributable ASR of 28.3 per 100,000 (Table 4).

### Vulvar Cancer and HPV

There were 25 vulvar cancer cases reported by the PBCRs from 2012 to 2014 representing 1.8% of all female cancers and an ASR of 1.3 per 100,000 (Table 3). About half of the vulvar cancer cases were seen in the 45 to 54 years age group. Using PAF of 40% from a meta-analysis (18), 10 of the 25 cases were attributable to HPV infection yielding an attributable ASR of 0.5 per 100,000 (Table 4).

### Vaginal Cancer and HPV

Eight vaginal cancer cases were reported by both registries, constituting 0.6% of female cancers in the study period with an ASR of 0.4 per 100,000 (Table 3). Three to six of the eight vaginal cancer cases were attributable to HPV infection, using a PAF of 40–70% (13, 18). The ASR for vaginal cancers attributable to HPV infection ranged from 0.2 to 0.3 per 100,000 (Table 4).

### Anal Cancer and HPV

There were 38 anal cancer cases reported by the ABCR and ECR from 2012 to 2014. Seventeen cases were reported in females, representing 1.3% of female cancers and 21 cases reported in males, accounting for 2.7% of male cancers (Table 3). The combined ASR was 1.1 per 100,000 with ASR of 0.7 per 100,000 for females and 1.4 per 100,000 for males (Table 3). In our study, anal cancer was the most common HPV-associated cancer in males. Most of the anal cancer cases were seen between the age groups 35 and 54 years (19 cases) in both sexes. Using a PAF of 88 to 90% (13, 19), we estimated that 33 anal cancers in both sexes were attributable to HPV. The combined ASR for anal cancers attributable to HPV infection was 0.9 per 100,000; 1.2 per 100,000 in males and 0.6 per 100,000 in females (Table 4).

### Oropharyngeal Cancer and HPV

Some 23 oropharyngeal cancer (OPC) were reported by the registries from 2012 to 2014 with 8 cases in males, representing 1.9% of male cancers and 15 cases in females, constituting 0.6% of female cancers (Table 3). The combined ASR for OPC was 0.7 per 100,000, with 0.8 per 100,000 in males, and 0.5 per 100,000 females, respectively (Table 3). Using a PAF estimate of 12 to 46% (13, 21), we found that 3 to 11 of the 23 OPC in both sexes were attributable to HPV infection. The combined ASR for OPC attributable to HPV infection ranged from 0.1 to 0.4 per 100,000; 0.1 to 0.4 per 100,000 for males and 0.0 to 0.3 per 100,000 for females (Table 4).

### Penile Cancer and HPV

Two cases of penile cancers were reported by the registries. Of these two cases, one penile cancer case was attributable to HPV infection using a PAF of 48% (13, 23). The ASR for penile cancer attributable to HPV was 0.1 per 100,000 (Table 4).

### Bladder Cancer and Schistosoma

The two registries reported 44 cases of bladder cancers in the study period. More cases were reported in males (30 cases) compared to females (14 cases). The combined ASR was 1.3 per 100,000, with ASR of 1.4 per 100,000 and 1.1 per 100,000 in males and females, respectively. Using a PAF estimate of 56% (13), we estimated that 25 bladder cancer cases were attributable to Schistosoma infection, with combined ASR of 0.7 per 100,000; 0.8 per 100,000 in men and 0.6 per 100,000 in women (Table 4).

### Liver Cancer and HBV/HCV

A total of 145 liver cancer cases were reported in both sexes from 2012 to 2014 by ABCR and ECR. Of these, 52 cases occurred in females and 93 cases were seen in males (Table 3). Liver cancer was the most common infections-associated cancer in males, representing 11.9% of male cancers. In females, liver cancer was the second most common infections-associated cancer constituting 3.9% of all female cancers reported by the PBCRs. The combined ASR for liver cancer was 3.4 per 100,000, with 4.0 per 100,000 in males and 2.7 per 100,000 in females, respectively (Table 3). Liver cancer was most commonly seen in the 25 to 34 and 35 to 44 years age groups with 37 cases, each in females. In males, liver cancer cases were commoner among the 25 to 34 years age group with 23 cases. Using the PAF estimate of 92% (13), we estimated that 134 cases of the 145 liver cancer cases reported were attributable to HBV/HCV infection with combined ASR of 3.1 per 100,000; 2.5 per 100,000 in males and 3.7 per 100,000 in females (Table 4).

### Stomach Cancer and *Helicobacter pylori*

There were 56 cases of stomach cancers reported in our study with 23 cases in females and 33 cases in males (Table 3). Stomach cancer accounted for 1.7% of all female cancers and 4.4% of all male cancers reported by the ABCR and ECR from 2012 to 2014 (Table 3). The combined ASR was 1.6 per 100,000, with 1.4 per 100,000 in females and 1.7 per 100,000 in males. Stomach cancer was more common among the 45 to 54 years age groups in both sexes, with 13 cases in females and 11 cases in males. Using a PAF estimate of 74% (13), we estimated that 41 cases of the 56 cases of stomach cancer were attributable to *H. pylori* infection, with combined ASR of 1.1 per 100,000; 1.2 per 100,000 in males and 1.0 per 100,000 in females (Table 4).

### Nasopharyngeal Cancer and EBV

The PBCRs reported 81 nasopharyngeal cancer cases in both sexes from 2012 to 2014. Thirty-four cancer cases were seen in females, representing 2.5% of female cancers and 47 cancer cases in males, constituting 6.1% of male cancers (Table 3). The combined ASR was 1.6 per 100,000, with 1.5 per 100,000 in females and 1.7 per 100,000 in males. Nasopharyngeal cancer was more common in males in the 45 to 54 years age group with 18 cases, while in females, it was most common among the 45 to 54 and 55 to 64 years age groups with 13 and 14 cases, respectively. We used a PAF estimate of 100% because EBV has been shown to be a necessary cause of nasopharyngeal cancer (13, 28) and assumed that all 81 nasopharyngeal cancers were attributable to EBV infection (Table 3). The combined ASR for nasopharyngeal cancers attributable to EBV infection was 1.6 per 100,000; 1.7 per 100,000 in males and 1.5 per 100,000 females (Table 4).

### Hodgkin Lymphoma and EBV

Some 45 HL cases were reported by ABCR and ECR within the study period. Of these, 17 cases occurred in females and 28 cases in males (Table 3). The combined ASR was 0.8 per 100,000, with 0.5 per 100,000 in females and 1.0 per 100,000 in males. The highest numbers of HL cases were reported in the 25 to 34 years age groups in both sexes with 13 cases. In our study, we calculated the EBV-attributable fraction for HL using a PAF estimate of 80% from a study by Lynnette et al. and a meta-analysis (29, 30). Thirty-six of the 45 HL cases reported by the PBCRs were attributable to EBV. The combined ASR for HL attributable to EBV infection was 1.0 per 100,000; 0.4 per 100,000 in females and 1.4 per 100,000 in males (Table 4).

### Non-Hodgkin Lymphoma and EBV/HIV

The registries reported 110 cases of NHL from 2012 to 2014. Forty-nine cases were seen in females and 61 cases in males (Table 3). NHL was the second most common infection-associated cancer reported in males. The combined ASR was 5.0 per 100,000, with 2.3 per 100,000 in females and 2.7 per 100,000 in males. We used a PAF of 100% as sero-epidemiological studies have shown a strong association of NHL with EBV (13). We estimated that 110 cases reported by the registries were attributable to EBV and HIV, with combined ASR of 2.5 per 100,000; 2.3 per 100,000 in females and 2.7 per 100,000 in males (Table 4).

### Kaposi Sarcoma and HIV/HHV8

The combined ASR for KS reported by the PBCR was 2.4 per 100,000, with 1.1 per 100,000 in males and 1.3 per 100,000 in females (Table 3). There were 61 cases reported from 2012 to 2014. Of these, 26 cases occurred in females representing 2.0% of all female cancers, while 28 cases were seen in males accounting for 4.6% of all male cancers (Table 3). We found the highest number of KS cases in both sexes in the 35 to 44 years and 45 to 54 years age groups with 24 cases each. We used a PAF estimate of 100% in our study because studies have shown that HHV8 and KS in HIV positive individuals is an AIDS defining cancer (32, 36). Therefore, we assume that all 61 cases of KS reported by the PBCRs in 2012–2014 were attributable to HIV/HHV8 infection with combined ASR of 1.2 per 100,000, with 1.1 per 100,000 in males and 1.3 per 100,000 in females (Table 4).

## Discussion

In this study, we found that almost a quarter of cancer cases (23.8%) in these two PBCR were associated with infections and most of these (22.0% of new cancer cases and 92% of the infections-associated cancers) were indeed attributable to infections. This is similar to estimates of 22.9% for other developing countries; higher than 7.4% estimated for developed countries and expectedly higher than global average of 16.1% reported by de Martel et al in 2008 (1).

Human papillomavirus infection is one of the most important infections-associated with cancers worldwide (37). In 2008, 700,000 of the 2 million cases of infections-associated cancers occurred at sites, such as cervix uteri, anus, penis, vulva, vagina, and oropharynx where HPV infections are prevalent. Some 610,000 of these cancers were attributable to HPV infection and 490,000 (80.4%) were estimated to have occurred in developing countries (37). HPV-associated cancers were the commonest infections-associated cancer in this study. This contrasts with the situation in more developed regions of the world and Northeast Asia where *H. pylori* infection is the most common infections-associated with cancers (1, 7).

Cervical cancer was the most common infections-associated cancer in this study and is the second commonest cancer in Nigerian women (10). The ASR for cervical cancer in this study was 28.3 per 100,000 and that is similar to estimates for West-Africa (ASR of 29.3 per 100,000) (38) and SSA that ranges from 20 to 35 per 100,000 (11). Early detection measures are particularly relevant for cervical cancer and population-wide screening programs if implemented alongside vaccination with HPV would significantly reduce incidence and mortality from cervical cancer and other HPV-associated cancers in Nigeria.

*Helicobacter pylori* infection is associated with stomach cancers but these are relatively rare in the Nigerian population. The prevalence of stomach cancers in SSA is low with the lowest incidence rates reported in Western Africa (39), somewhat higher rates in Eastern Africa (40) while the highest rates globally are seen in Eastern Asia (41). However, given that patients with dyspeptic symptoms suspicious of stomach cancer may not be adequately investigated due to paucity of endoscopy and biopsy services in this environment, the incidence of stomach cancers may be under-reported (42).

In our study, liver cancer was the most common infections-associated cancer in males. Over 75% of liver cancers are hepatocellular carcinomas (HCC) that are largely due to infection with HBV and HCV (43), which are endemic in SSA (44, 45). There is wide variation in liver cancer incidence rates worldwide due to geographic variation in prevalence of chronic HBV and HCV infections (43), aflatoxin exposure (46), alcohol-related cirrhosis (47), fatty liver disease (48), obesity, and smoking (43). In our study, we found ASR of 4.0 per 100,000 in males and 1.1 per 100,000 in females with a rate ratio of 3.6, similar to findings from The Gambia of 1.6 per 100,000 in 2009 (49). Somewhat higher incidence rates have been reported from Zimbabwe, 14.4 per 100,000 and 12.7 per 100,000 in males and females, respectively (rate ratio 1.1) and in Uganda 8.7 per 100,000 and 5.8 per 100,000 in males and females respectively (rate ratio 1.5) (43). The highest liver cancer incidence rates worldwide were reported in Eastern Asia although these rates appear to be declining in three of the seven countries included in the study namely China, the Philippines, and Japan (43).

Human papillomavirus (50) was the first virus shown to be associated with human cancer and is linked with several cancers, including nasopharyngeal cancers, HL, and NHL (13). In our study, the ASR of nasopharyngeal cancers was 1.7 per 100,000 in males and 1.5 per 100,000 in females. Although nasopharyngeal cancers are rare in many populations, high incidence rates have been reported in Northern Africa (51). Other risk factors for nasopharyngeal cancers include consumption of salt-preserved fish, positive family history, tobacco smoking, and a history of chronic respiratory tract conditions (52). Protective factors include consumption of fruits and vegetables and possession of some human leukocyte antigen (HLA) genotypes that may reduce risk (53). NHL is an AIDS defining cancer. However, the availability of antiretroviral therapy has largely led to a reduction in the risk of NHL in HIV-infected individuals in recent times (31).

The increasing availability of antiretroviral therapy has contributed to the reduction in the incidence of KS among HIV positive individuals in developing countries. KS is an AIDS defining cancer that is linked to HIV and HHV8 infections (53). Evidence of a strong association exists between KS and immunocompromised individuals because KS risk increases as immune function is impaired in HIV-infected individuals (54). The prevalence of HHV8 is higher in HIV-infected men who have sex with men (MSM) than in heterosexual HIV-infected individuals (53). The incidence rates of KS in our study were 1.1 in males and 1.3 in females per 100,000. These low rates were similar to findings by Bah et al. in The Gambia (55), and significantly lower than incidence rates reported from Zimbabwe 47.2 per 100,000 and Uganda 39.3 per 100,000 (55).

Our study has several important limitations. We used cancer registry data from two PBCRs in Nigeria because the two other PBCR have just been established and did not have data covering the same period. While we strive to ensure high quality of cancer registration in Nigeria, our data may be incomplete given the high proportion of morphologically verified cases in the databases of the registries that we used. There may be under-reporting of cancers that are hard to biopsy because of limited diagnostic facilities, and cost of diagnosis and treatment. Another limitation is the small sample sizes of the few prevalence studies of infectious agents in Nigeria, which necessitated the use of data from meta-analyses to estimate our PAF. These estimates may not be a true representation of the Nigerian population. We did not estimate the incidence of certain cancers, such as acute T-cell leukemia/lymphoma which is associated with HTLV-1 or bile duct cancer which is associated with *C. sinensis* and *O. viverrini* because few of these cancers were reported by the registries.

In our sensitivity analyses, we found that 35.9% of the Nigerian cancer cases reported in GLOBOCAN 2012 database were associated with infections and 33.8% were attributable to infections. The differences in proportion of cancers attributable to infections from our PBCRs and GLOBOCAN 2012 data for Nigeria could be due to the fact that the GLOBOCAN 2012 database contained incidence estimates for only 7 out of the 12 infections-associated cancers that we considered. Our findings also shows that liver cancer was either over-estimated in GLOBOCAN 2012 or under-reported by the PBCRs whose data we analyzed in this study. GLOBOCAN 2012 reported ASR of 7.5 per 100 000 for liver cancer in females and 13.8 per 100,000 in males, while we found ASR of 2.5 per 100,000 in females and 3.7 per 100,000 in males.

## Conclusion

Despite these limitations, our study confirms the crucial role that infections plays in the etiology of a wide range of cancers in the Nigerian population and the opportunities for reducing morbidity and mortality through control of these infections. Prophylactic vaccination against HPV and HBV, and effective management of *H. pylori* and HCV infections offer tremendous opportunities to significantly reduce the burden of infections-attributable cancers in Nigeria.

## Author Contributions

MO, EJ-A, ED, and EO contributed to data collection, data analyses, and drafting the manuscript. All authors contributed to the writing of this manuscript. FI, TO, EE, and RH contributed to data collection and data quality. CA conceived the idea for the study, obtained funding, provided critical revisions, and guided all aspects of the paper. All authors read and approved the final manuscript.

## Conflict of Interest Statement

The authors declare that the research was conducted in the absence of any commercial or financial relationships that could be construed as a potential conflict of interest.

## References

[1] De Martel C Ferlay J Franceschi S Vignat J Bray F Forman D Global burden of cancers attributable to infections in 2008: a review and synthetic analysis. Lancet Oncol (2012) 13:607–15. 10.1016/s1470-2045(12)70137-7 22575588

[2] International Agency for Research on Cancer. IARC Monograph on Biological Agents Volume 100B: A Review of Human Carcinogens. Lyon: IARC Working Group (2012). p. 1–441.

[3] Whiteman DC Webb PM Green AC Neale RE Fritschi L Bain CJ Cancers in Australia in 2010 attributable to modifiable factors: summary and conclusions. Aust N Z J Public Health (2015) 39:477–84. 10.1111/1753-6405.12471 26437735 PMC4606779

[4] Parkin DM Cancers attributable to infection in the UK in 2010. Br J Cancer (2011) 105(Suppl 2):S49–56. 10.1038/bjc.2011.484 22158321 PMC3252069

[5] van Lier EA van Kranen HJ van Vliet JA Rahamat-Langendoen JC. Estimated number of new cancer cases attributable to infection in the Netherlands in 2003. Cancer Lett (2008) 272:226–31. 10.1016/j.canlet.2008.07.007 18752887

[6] Ortiz AP Soto-Salgado M Calo WA Tortolero-Luna G Perez CM Romero CJ Incidence and mortality rates of selected infection-related cancers in Puerto Rico and in the United States. Infect Agent Cancer (2010) 5:10. 10.1186/1750-9378-5-10 20470399 PMC2891681

[7] Shin A Park S Shin HR Park EH Park SK Oh JK Population attributable fraction of infection-related cancers in Korea. Ann Oncol (2011) 22:1435–42. 10.1093/annonc/mdq592 20974652

[8] Boffetta P Tubiana M Hill C Boniol M Aurengo A Masse R The causes of cancer in France. Ann Oncol (2009) 20:550–5. 10.1093/annonc/mdn597 18765462

[9] Xiang W Shi JF Li P Wang JB Xu LN Wei WQ Estimation of cancer cases and deaths attributable to infection in China. Cancer Causes Control (2011) 22:1153–61. 10.1007/s10552-011-9791-y 21667067

[10] Jedy-Agba E Curado MP Ogunbiyi O Oga E Fabowale T Igbinoba F Cancer incidence in Nigeria: a report from population-based cancer registries. Cancer Epidemiol (2012) 36:e271–8. 10.1016/j.canep.2012.04.007 22621842 PMC3438369

[11] International Agency for Research on Cancer. GLOBOCAN 2012. Lyon: IARC Cancer Base (2016).

[12] Jedy-Agba E Ezeome E Oga E Odutola M Okoroafor A Hassan R Cancer incidence in South-Eastern Nigeria-first results from the Enugu cancer registry. Asia Pac J Clin Oncol (2014) 10:2018.

[13] Parkin DM. The global health burden of infection-associated cancers in the year 2002. Int J Cancer (2006) 118:3030–44. 10.1002/ijc.21731 16404738

[14] Denny L Adewole I Anorlu R Dreyer G Moodley M Smith T Human papillomavirus prevalence and type distribution in invasive cervical cancer in sub-Saharan Africa. Int J Cancer (2014) 134:1389–98. 10.1002/ijc.28425 23929250

[15] Attoh S Asmah R Wiredu EK Gyasi R Tettey Y. Human papilloma virus genotypes in Ghanaian women with cervical carcinoma. East Afr Med J (2010) 87:345–9. 23451558

[16] Bosch FX Lorincz A Munoz N Meijer CJ Shah KV. The causal relation between human papillomavirus and cervical cancer. J Clin Pathol (2002) 55:244–65. 10.1136/jcp.55.4.244 11919208 PMC1769629

[17] Tesfalul M Simbiri K Wheat CM Motsepe D Goldbach H Armstrong K Oncogenic viral prevalence in invasive vulvar cancer specimens from human immunodeficiency virus-positive and -negative women in Botswana. Int J Gynecol Cancer (2014) 24:758–65. 10.1097/igc.0000000000000111 24651632 PMC3999226

[18] De Vuyst H Clifford GM Nascimento MC Madeleine MM Franceschi S. Prevalence and type distribution of human papillomavirus in carcinoma and intraepithelial neoplasia of the vulva, vagina and anus: a meta-analysis. Int J Cancer (2009) 124:1626–36. 10.1002/ijc.24116 19115209

[19] Alemany L Saunier M Alvarado-Cabrero I Quiros B Salmeron J Shin HR Human papillomavirus DNA prevalence and type distribution in anal carcinomas worldwide. Int J Cancer (2015) 136:98–107. 10.1002/ijc.28963 24817381 PMC4270372

[20] Kaba G Dzudzor B Gyasi RK Asmah RH Brown CA Kudzi W Human papillomavirus genotypes in a subset of head and neck squamous cell carcinoma. West Afr J Med (2014) 33:121–4. 25236828

[21] Ndiaye C Mena M Alemany L Arbyn M Castellsague X Laporte L HPV DNA, E6/E7 mRNA, and p16INK4a detection in head and neck cancers: a systematic review and meta-analysis. Lancet Oncol (2014) 15:1319–31. 10.1016/s1470-2045(14)70471-1 25439690

[22] Senba M Buziba N Mori N Wada A Irie S Toriyama K Detection of human papillomavirus and cellular regulators p16INK4a, p53, and NF-κB in penile cancer cases in Kenya. Acta Virol (2009) 53:43–8. 10.4149/av_2009_01_43 19301950

[23] Backes DM Kurman RJ Pimenta JM Smith JS. Systematic review of human papillomavirus prevalence in invasive penile cancer. Cancer Causes Control (2009) 20:449–57. 10.1007/s10552-008-9276-9 19082746

[24] Sylvester C Liver cancer in Enugu, South East Nigeria. Insight Bioinforma (2011) 1:1–5. 10.5567/BIOINFO-IK.2011.1.5

[25] Kirk GD Lesi OA Mendy M Akano AO Sam O Goedert JJ The Gambia liver cancer study: infection with hepatitis B and C and the risk of hepatocellular carcinoma in West Africa. Hepatology (2004) 39:211–9. 10.1002/hep.20027 14752840

[26] Udoh MO Obaseki DE. Histopathological evaluation of *H. pylori* associated gastric lesions in Benin city, Nigeria. East Afr Med J (2012) 89:408–13. 26852453

[27] Newton R Ziegler JL Casabonne D Carpenter L Gold BD Owens M *Helicobacter pylori* and cancer among adults in Uganda. Infect Agent Cancer (2006) 1:5. 10.1186/1750-9378-1-5 17150134 PMC1660530

[28] Coghill AE Hildesheim A. Epstein-Barr virus antibodies and the risk of associated malignancies: review of the literature. Am J Epidemiol (2014) 180:687–95. 10.1093/aje/kwu176 25167864 PMC4271109

[29] Iliyasu Y Ayers LW Liman AA Waziri GD Shehu SM. Epstein-Barr virus association with malignant lymphoma subgroups in Zaria, Nigeria. Niger J Surg Sci (2014) 23:6–8. 10.4103/1116-5898.127096 25620871 PMC4302412

[30] Tumwine LK Orem J Kerchan P Byarugaba W Pileri SA. EBV, HHV8 and HIV in B cell non Hodgkin lymphoma in Kampala, Uganda. Infect Agent Cancer (2010) 5:12. 10.1186/1750-9378-5-12 20591151 PMC2907314

[31] Jaquet A Odutola M Ekouevi DK Tanon A Oga E Akakpo J Cancer and HIV infection in referral hospitals from four West African countries. Cancer Epidemiol (2015) 39:1060–5. 10.1016/j.canep.2015.09.002 26375806 PMC4679441

[32] Schulz TF. Kaposi’s sarcoma-associated herpesvirus (human herpesvirus 8): epidemiology and pathogenesis. J Antimicrob Chemother (2000) 45(Suppl T3):15–27. 10.1093/jac/45.suppl_4.15 10855768

[33] Sitas F Carrara H Beral V Newton R Reeves G Bull D Antibodies against human herpesvirus 8 in black South African patients with cancer. N Engl J Med (1999) 340:1863–71. 10.1056/nejm199906173402403 10369849

[34] Walboomers JM Jacobs MV Manos MM Bosch FX Kummer JA Shah KV Human papillomavirus is a necessary cause of invasive cervical cancer worldwide. J Pathol (1999) 189:12–9. 10.1002/(sici)1096-9896(199909)189:1<12:aid-path431>3.0.co;2-f 10451482

[35] Oga EA Schumaker LM Alabi BS Obaseki D Umana A Bassey IA Paucity of HPV-related head and neck cancers (HNC) in Nigeria. PLoS One (2016) 11:e0152828. 10.1371/journal.pone.0152828 27050815 PMC4822856

[36] Okuku F Omoding A Walusansa V Origa M Mutungi G Orem J. Infection-related cancers in sub-Saharan Africa: a paradigm for cancer prevention and control. Oncology (2013) 84:75–80. 10.1159/000343151 23128067

[37] Forman D de Martel C Lacey CJ Soerjomataram I Lortet-Tieulent J Bruni L Global burden of human papillomavirus and related diseases. Vaccine (2012) 30(Suppl 5):F12–23. 10.1016/j.vaccine.2012.07.055 23199955

[38] Louie K Sanjose S Mayaud P. Epidemiology and prevention of human papillomavirus and cervical cancer in sub-Saharan Africa: a comprehensive review. Trop Med Int Health (2009) 14:1287–302. 10.1111/j.1365-3156.2009.02372.x 19772550

[39] Parkin DM. Global cancer statistics in the year 2000. Lancet Oncol (2001) 2:533–43. 10.1016/s1470-2045(01)00486-7 11905707

[40] Wabinga H. *Helicobacter pylori* and histopathological changes of gastric mucosa in Uganda population with varying prevalence of stomach cancer. Afr Health Sci (2005) 5:234–7. 10.5555/afhs.2005.5.3.234 16245994 PMC1831938

[41] Rahman R Asombang AW Ibdah JA. Characteristics of gastric cancer in Asia. World J Gastroenterol (2014) 20:4483–90. 10.3748/wjg.v20.i16.4483 24782601 PMC4000485

[42] Ahmed A Ukwenya AY Makama JG Mohammad I Management and outcome of gastric carcinoma in Zaria, Nigeria. Afr Health Sci (2011) 11:353–61. 22275924 PMC3261017

[43] Center MM Jemal A. International trends in liver cancer incidence rates. Cancer Epidemiol Biomarkers Prev (2011) 20:2362–8. 10.1158/1055-9965.epi-11-0643 21921256

[44] Ndububa DA Ojo OS Adetiloye VA Durosinmi MA Olasode BJ Famurewa OC Chronic hepatitis in Nigerian patients: a study of 70 biopsy-proven cases. West Afr J Med (2005) 24:107–11. 16092308 10.4314/wajm.v24i2.28177

[45] Layden JE Phillips R Opare-Sem O Akere A Salako BL Nelson K Hepatitis C in sub-Saharan Africa: urgent need for attention. Open Forum Infect Dis (2014) 1:ofu065. 10.1093/ofid/ofu065 25734135 PMC4281810

[46] Liu Y Wu F. Global burden of aflatoxin-induced hepatocellular carcinoma: a risk assessment. Environ Health Perspect (2010) 118:818–24. 10.1289/ehp.0901388 20172840 PMC2898859

[47] Mazzanti R Arena U Tassi R Hepatocellular carcinoma: where are we? World J Exp Med (2016) 6:21–36. 10.5493/wjem.v6.i1.21 26929917 PMC4759352

[48] Yu J Marsh S Hu J Feng W Wu C. The pathogenesis of nonalcoholic fatty liver disease: interplay between diet, gut microbiota, and genetic background. Gastroenterol Res Pract (2016) 2016:2862173. 10.1155/2016/2862173 27247565 PMC4876215

[49] Bah E Carrieri MP Hainaut P Bah Y Nyan O Taal M. 20-Years of population-based cancer registration in hepatitis B and liver cancer prevention in the Gambia, West Africa. PLoS One (2013) 8:e75775. 10.1371/journal.pone.0075775 24098724 PMC3787012

[50] Lefebvre L Sol D. Brains, lifestyles and cognition: are there general trends? Brain Behav Evol (2008) 72:135–44. 10.1159/000151473 18836259

[51] Jemal A Bray F Forman D O’Brien M Ferlay J Center M Cancer burden in Africa and opportunities for prevention. Cancer (2012) 118:4372–84. 10.1002/cncr.27410 22252462

[52] Chang ET Adami HO. The enigmatic epidemiology of nasopharyngeal carcinoma. Cancer Epidemiol Biomarkers Prev (2006) 15:1765–77. 10.1158/1055-9965.epi-06-0353 17035381

[53] Lodi S Guiguet M Costagliola D Fisher M de Luca A Porter K Kaposi sarcoma incidence and survival among HIV-infected homosexual men after HIV seroconversion. J Natl Cancer Inst (2010) 102:784–92. 10.1093/jnci/djq134 20442214 PMC2879418

[54] Mbulaiteye SM Biggar RJ Goedert JJ Engels EA. Immune deficiency and risk for malignancy among persons with AIDS. J Acquir Immune Defic Syndr (2003) 32:527–33. 10.1097/00126334-200304150-00010 12679705

[55] Bah E Parkin DM Hall AJ Jack AD Whittle H Cancer in the Gambia: 1988-97. Br J Cancer (2001) 84:1207 10.1054/bjoc.2001.1730 11336472 PMC2363873

